# Black pleural effusion caused by a pancreaticopleural fistula associated with autoimmune pancreatitis: A case report

**DOI:** 10.1097/MD.0000000000030322

**Published:** 2022-09-09

**Authors:** Keiki Miyadera, Kakeru Hisakane, Yuki Kato, Kenichiro Atsumi, Hiroki Ono, Shu Tanaka, Kaoru Kubota, Masahiro Seike, Akihiko Gemma, Takashi Hirose

**Affiliations:** a Department of Pulmonary Medicine and Medical Oncology, Nippon Medical School Tamanagayama Hospital, Tokyo, Japan; b Department of Gastroenterology, Nippon Medical School Tamanagayama Hospital, Tokyo, Japan; c Department of Pulmonary Medicine and Oncology, Graduate School of Medicine, Nippon Medical School, Tokyo, Japan.

**Keywords:** autoimmune pancreatitis, black pleural effusion, pancreaticopleural fistula

## Abstract

Black pleural effusion is a rare medical condition and a diagnostic marker. Pancreaticopleural fistula is one of the causes of black pleural effusion. Thus far, black pleural effusions caused by pancreaticopleural fistulae have mostly been reported in patients with alcohol-induced chronic pancreatitis. In this report, we present a case of black pleural effusion caused by a pancreaticopleural fistula associated with autoimmune pancreatitis.

**Patient concerns and diagnosis:**

A 59-year-old female without a history of alcohol drinking presented to our hospital with a chief complaint of dyspnea, as well as chest and back discomfort. She had left pleural effusion, and thoracentesis showed black pleural effusion. Computed tomography revealed the presence of encapsulated fluid from the pancreatic tail to the left pleural cavity, which was diagnosed as a pancreaticopleural fistula. It also showed diffuse pancreatic swelling. Serum testing showed a high IgG4 level (363 mg/dL). These findings led to the diagnosis of autoimmune pancreatitis.

**Interventions and outcome:**

The patient underwent endoscopic pancreatic sphincterotomy and pancreatic duct stent placement and received treatment with steroids. After treatment, there was no further accumulation of pleural effusion observed.

**Conclusion:**

This is the first report of black pleural effusion due to a pancreaticopleural fistula associated with autoimmune pancreatitis. The characteristic appearance of black pleural effusion may assist diagnosis. We report this case to emphasize that autoimmune pancreatitis can be a cause of black pleural effusion.

## 1. Introduction

Black pleural effusion is a very rare condition with a characteristic appearance, which may be helpful in diagnosis.^[1]^ The presence of a pancreaticopleural fistula associated with a pancreatic pseudocyst may cause black pleural effusion.^[2]^ Thus far, most reports of black pleural effusion caused by a pancreaticopleural fistula have been associated with alcohol-induced pancreatitis.^[2–9]^ In this article, we report a case of pancreaticopleural fistula due to autoimmune pancreatitis, which was diagnosed based on the presence of black pleural effusion.

## 2. Case presentation

**Figure 1.**
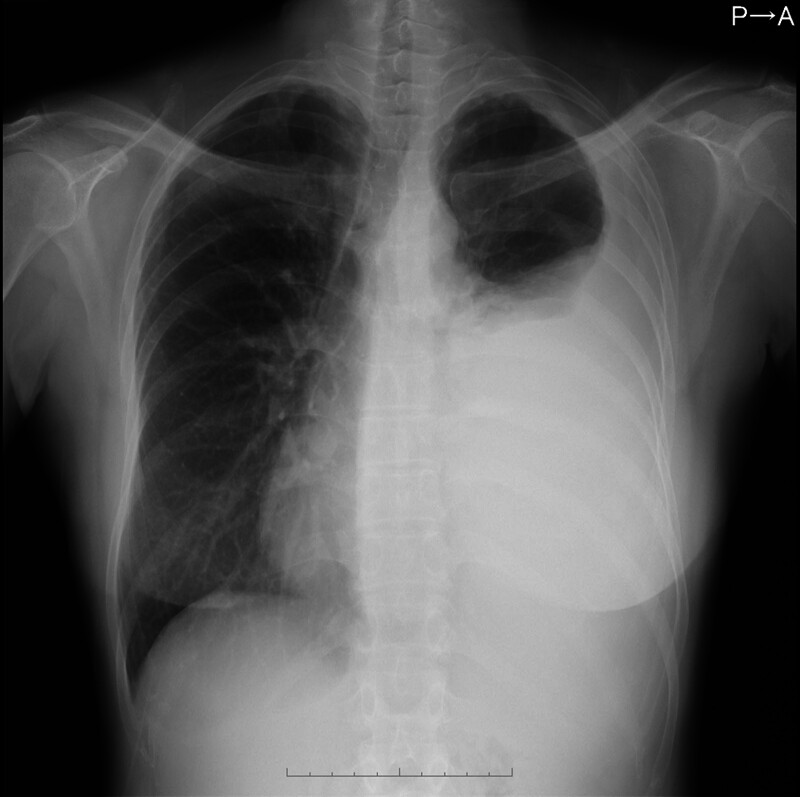
Chest radiograph showing massive left pleural effusion.

**Figure 2.**
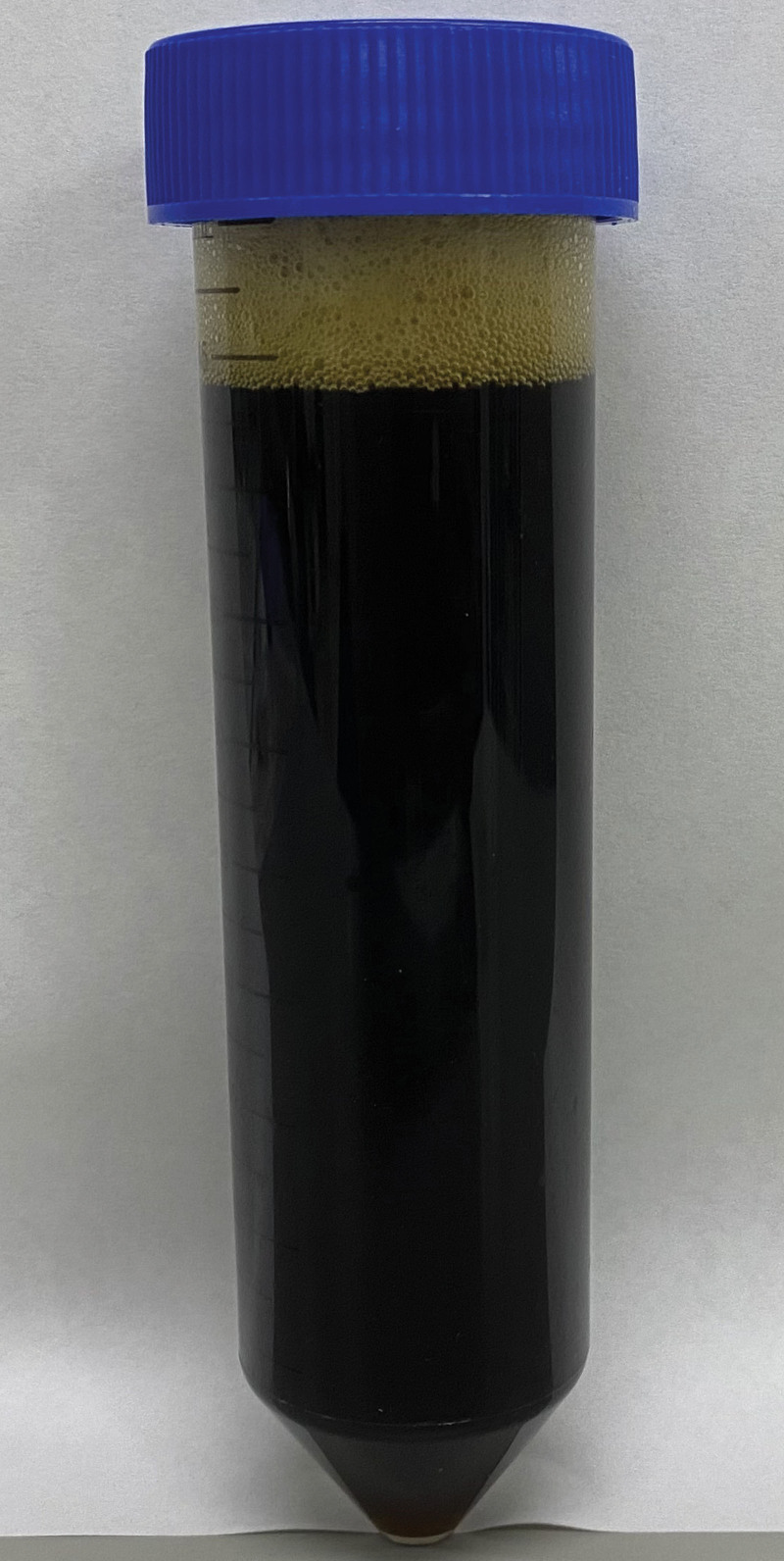
Black pleural effusion obtained by thoracentesis.

**Figure 3.**
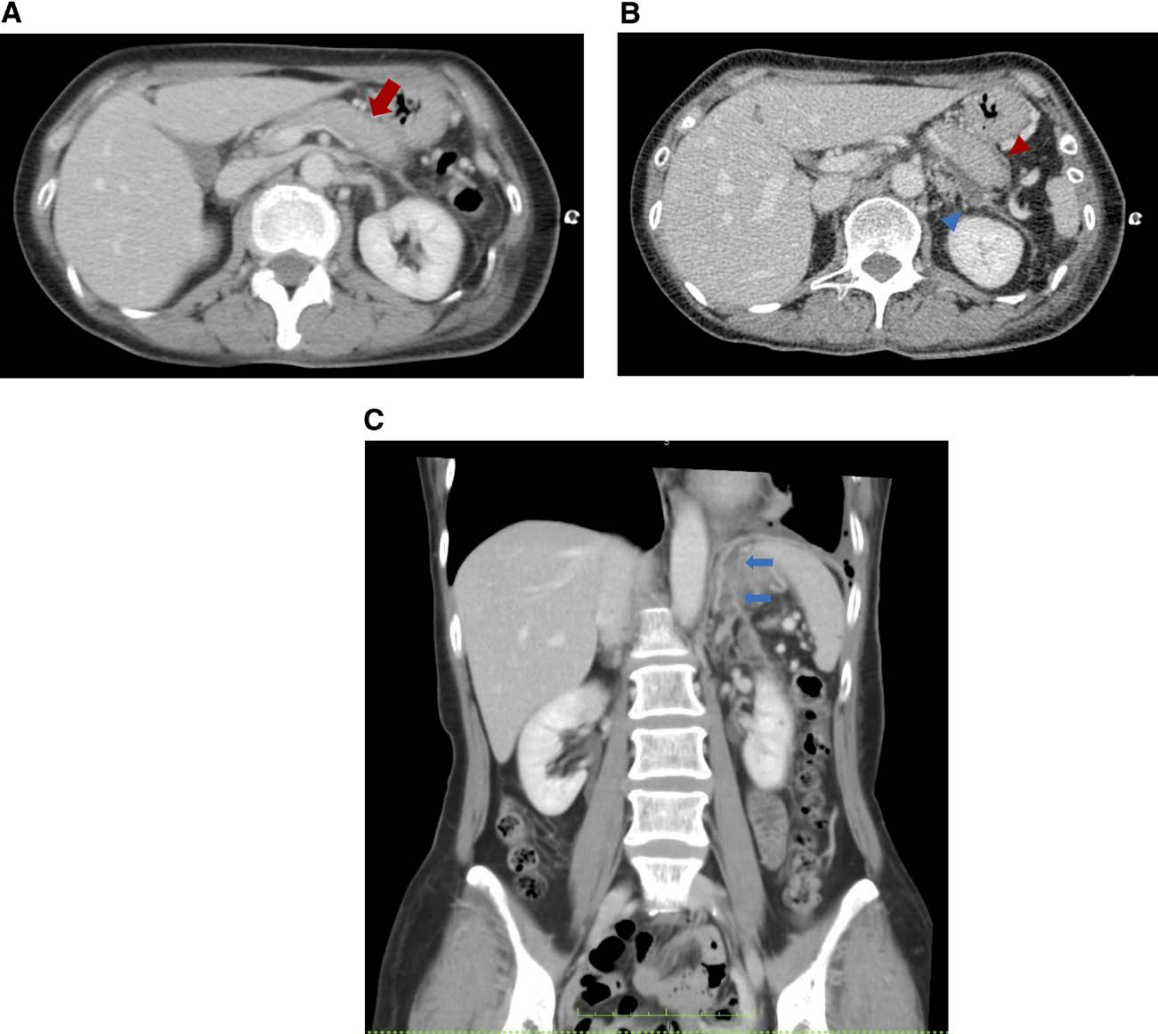
A, Thoracoabdominal contrast-enhanced CT showing diffuse enlargement of the pancreas (red arrow). B, A cyst with encapsulated fluid in the pancreatic tail (blue arrowhead) and a capsule-like rim around the pancreas (red arrowhead). C, Encapsulated fluid from the pancreatic tail to the left pleural cavity (blue arrows). CT, computed tomography.

**Figure 4.**
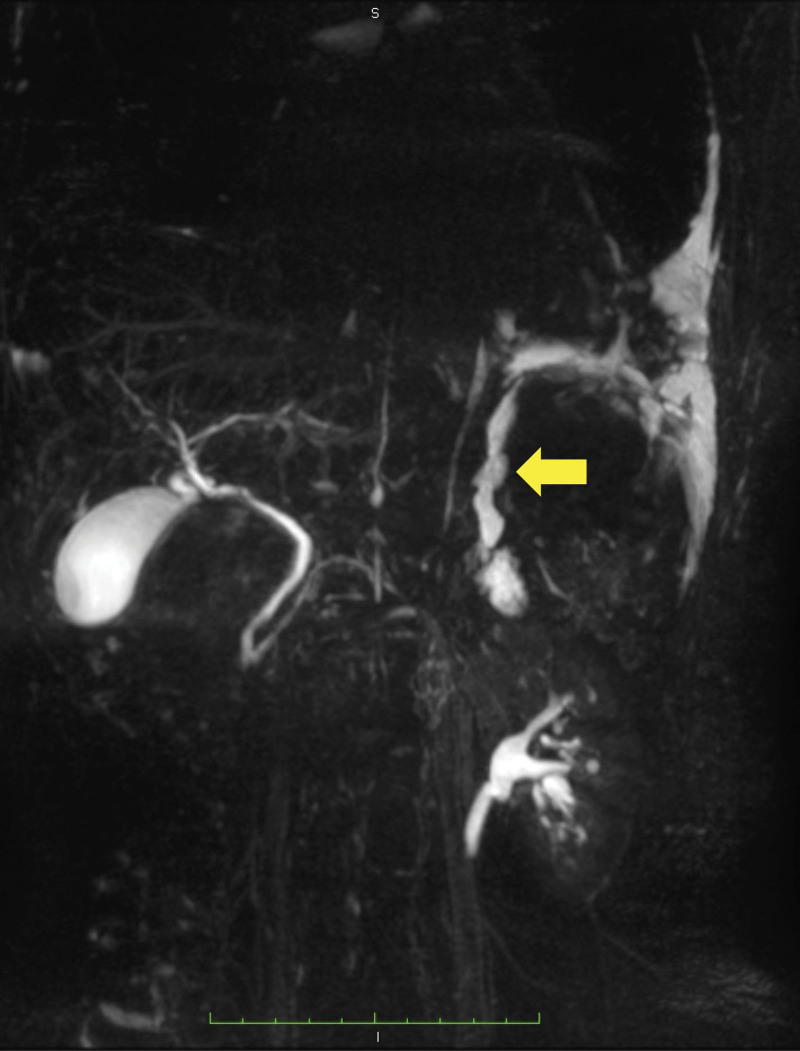
MRCP showing the presence of fluid from the pancreatic tail to the left thoracic cavity (yellow arrow). MRCP = magnetic resonance cholangiopancreatography.

A 59-year-old Japanese female presented to our hospital with dyspnea and discomfort in her chest and back for 1 month. Her medical history included only reflux esophagitis. She reported limited consumption of alcohol and did not have a smoking history. Her vital signs were normal. Physical examination showed diminished left lung sounds, absence of a cardiac murmur, and no abdominal abnormality. Laboratory data revealed high levels of amylase (228 U/L) and IgG4 (363 mg/dL), as well as positivity for antinuclear antibody (1:160). The chest radiograph revealed massive left pleural effusion (Fig. 1). Chest computed tomography (CT) showed massive left pleural effusion without any mass or enlarged mediastinal lymph nodes. The color of the pleural effusion was black, as observed following a puncture (Fig. 2). Examination of the pleural effusion revealed an exudative effusion with high levels of lactate dehydrogenase (778 IU/L), total protein (5.4 g/dL), amylase (10,543 IU/L), total bilirubin (4.9 mg/dL), indirect bilirubin (4.6 mg/dL), and iron (276 μg/dL). The amylase detected in the pleural effusion consisted of 100% pancreatic amylase. Cytological analysis of the pleural effusion and histological examination of the cellblock were negative for malignancy. The results of pleural effusion cultures were negative for bacteria, fungi, and acid-fast bacilli. She was admitted to our hospital for further examination and treatment. She underwent chest drainage for the left pleural effusion using a chest drainage tube. Thoracoabdominal contrast-enhanced CT after drainage showed diffuse enlargement of the pancreas, a capsule-like rim around the pancreas, the presence of a cystic lesion in the pancreatic tail, and encapsulated fluid from the pancreatic tail to the left pleural cavity (Figs. 3A–C). Magnetic resonance cholangiopancreatography also showed the presence of fluid from the pancreatic tail to the left pleural cavity (Fig. 4). She was diagnosed with autoimmune pancreatitis based on the findings of diffuse enlargement of the pancreas and a capsule-like rim, and the IgG4 level was ≥ 135 mg/dL. In addition, she was diagnosed with pancreaticopleural fistula based on the presence of the cystic lesion in the pancreatic tail, which was contiguous to the left pleural cavity. Based on the above findings, she was diagnosed with a pancreaticopleural fistula caused by a pancreatic pseudocyst linked to chronic autoimmune pancreatitis. We attempted to perform endoscopic pancreatic duct drainage; however, she refused blood transfusion for religious reasons, and we were unable to treat her further according to our hospital regulations. Therefore, she was transferred to another hospital for further treatment. She underwent endoscopic pancreatic sphincterotomy and pancreatic duct stent placement. Subsequently, she was treated with oral prednisolone (30 mg daily) for autoimmune pancreatitis. The pleural drain tube was removed after the initiation of steroid therapy, and she was discharged without recurrence of pleural effusion. Prednisolone has been gradually tapered to a maintenance dose of 5 mg daily. She has not had a recurrence of pleural effusion for 10 months after surgery.

## 3. Discussion

To our knowledge, this is the first report of black pleural effusion due to a pancreaticopleural fistula caused by autoimmune pancreatitis. Black pleural effusion is a rare condition; thus far, only 33 cases of black pleural effusion have been reported.^[10,11]^ Causes of black pleural effusion include malignant melanoma, pancreaticopleural fistula, fungal infections with *Aspergillus niger* and *Rhizopus oryzae*, lung cancer, benign malformation species, rheumatoid pleurisy, use of crack cocaine, and charcoal-containing empyema.^[12–14]^ Black pleural effusion due to a pancreaticopleural fistula is the consequence of intrathoracic hemorrhage and hemolysis by the pancreatic juice.^[2,3]^ In our case, the presence of red blood cells, as well as the high levels of indirect bilirubin (4.6 mg/dL) and iron (276 μg/dL) in the pleural effusion suggested that the cause was intrathoracic hemorrhage followed by hemolysis. To date, 9 cases of black pleural effusion caused by pancreaticopleural fistulae have been reported.^[10]^ Of those, 8 cases were caused by alcoholic pancreatitis, while the remaining case was induced by idiopathic pancreatitis.^[2–9,15]^ Almost all (99%) pancreaticopleural fistulae associated with a pancreatic pseudocyst are caused by alcoholic pancreatitis.^[16]^ Thus far, cases of pancreatic pseudocysts in autoimmune pancreatitis have been reported.^[17]^ But cases of black pleural effusion caused by pancreaticopleural fistulae due to autoimmune pancreatitis have not been reported. In our case, we diagnosed autoimmune pancreatitis based on CT findings and the high IgG4 level, and we also diagnosed pancreaticopleural fistula associated with autoimmune pancreatitis based on the presence of a pancreatic pseudocyst in the pancreatic tail and continuity into the left pleural cavity. The characteristic findings of black pleural effusion may lead to a diagnosis, and autoimmune pancreatitis should be considered as a differential diagnosis.

Treatment of black pleural effusion requires management of the underlying disease, such as chemotherapy for cancer or antifungal agents for fungal infections.^[10]^ In the present case, steroid therapy for autoimmune pancreatitis resolved the pleural effusion without recurrence. Patients with autoimmune pancreatitis receive steroid therapy, which is effective in many cases.^[18]^ Similarly, in our patient, steroid therapy was effective in controlling black pleural effusion due to autoimmune pancreatitis. She also underwent endoscopic pancreatic sphincterotomy and pancreatic duct stent placement. Previous cases of black pleural effusion caused by a pancreaticopleural fistula were treated using pancreatic duct stent placement (three cases), endoscopic nasopancreatic duct drainage (three cases), endoscopic ultrasound-guided drainage of a pancreatic pseudocyst (one case), and surgery (one case) (Table 1). Black pleural effusion may be controllable with appropriate treatment.

**Table 1:** Cases of black pleural effusion caused by a pancreaticopleural fistula.

Age (years)	Sex	Etiology	Treatment	Reference (first author, year)
54	Male	Alcohol	TD	Koide T,^[2]^ 2012
47	Female	Alcohol	TD	Huang TY,^[3]^ 2013
39	Male	Alcohol	TD and ENPD	Makino H,^[4]^ 2013
37	Female	Alcohol	TD and PDS	Kaur D,^[5]^ 2014
37	Female	Alcohol	TD, PDS, and octreotide	Mookherjee S,^[6]^ 2014
58	Male	Alcohol	TD, ENPD, and PDS	Hirosawa T,^[7]^ 2016
14	Female	Idiopathic	Surgery	Guo F,^[15]^ 2017
54	Male	Alcohol	TD and endoscopic pancreatic duct drainage	Ishigaki S,^[8]^ 2019
48	Male	Alcohol	TD and EUS-guided drainage	Arumairaj A,^[9]^ 2020

ENPD = endoscopic nasopancreatic drainage, EUS-guided drainage = endoscopic ultrasound-guided pancreatic pseudocyst drainage, PDS = pancreatic duct stent placement, TD = thoracic drainage.

Black pleural effusion is diagnosed by imaging followed by diagnostic thoracentesis for the analysis of its characteristics. In our case, the levels of amylase in the pleural effusion were elevated at 10,543 U/L. Elevated amylase in pleural effusion is also observed in acute pancreatitis, malignancy, and esophageal rupture; nevertheless, these levels are relatively low (≤1000 IU/L).^[2]^ In a report of pleural effusion associated with acute pancreatitis, the amylase levels were ≤ 4000 IU/L.^[19]^ In a study of 96 pancreaticopleural fistulae, the median value for the levels of amylase in the pleural effusion was 18,450 IU/L (range: 1830–164,187).^[19]^ High levels (>1000 IU/L) of amylase in the pleural effusion may be indicative of a pancreaticopleural fistula.^[1]^

## 4. Conclusion

This is the first report of black pleural effusion due to a pancreaticopleural fistula associated with autoimmune pancreatitis. Treatment of the underlying disease was also effective in resolving the black pleural effusion. This condition is a characteristic pleural finding and an important diagnostic marker. Pancreaticopleural fistula should be considered as a differential diagnosis for black pleural effusion. Moreover, it is essential to measure the levels of amylase through examination of the pleural effusion. In addition to alcohol-induced pancreatitis, autoimmune pancreatitis should be considered as a cause of pancreaticopleural fistulae.

## Acknowledgments

We thank Dr Ryuichi Yamamoto for treating the patient at the transfer hospital.

## Author Contributions

Conceptualization: Keiki Miyadera, Kakeru Hisakane, Takashi Hirose.

Investigation: Yuki Kato, Kenichiro Atsumi, Hiroki Ono, Shu Tanaka.

Supervision: Kaoru Kubota, Masahiro Seike, Akihiko Gemma, Takashi Hirose.

Writing – original draft: Keiki Miyadera.

Writing – review & editing: Kakeru Hisakane, Takashi Hirose.
